# Bioaerosol Exposures and Respiratory Diseases in Cannabis Workers

**DOI:** 10.1007/s11882-024-01157-7

**Published:** 2024-06-15

**Authors:** Tess Eidem, Tara Nordgren, Mark Hernandez

**Affiliations:** 1 Department of Civil, Environmental, and Architectural Engineering, University of Colorado Boulder, Boulder, CO 80309-0428 US; 2 Department of Environmental and Radiological Health Sciences, Colorado State University, Fort Collins, CO 80523-1601 US

**Keywords:** Cannabis Controlled Environment Agriculture (CEA), Cannabis Manufacturing, Bioaerosols, Occupational Respiratory Exposure, Microbial Inhalation Hazards, Aeroallergens

## Abstract

**Purpose of Review:**

This review investigates occupational inhalation hazards associated with biologically derived airborne particles (bioaerosols) generated in indoor cannabis cultivation and manufacturing facilities.

**Recent Findings:**

Indoor cannabis production is growing across the US as are recent reports of respiratory diseases among cannabis workers, including occupational asthma morbidity and mortality. More information is needed to understand how bioaerosol exposure in cannabis facilities impacts worker health and occupational disease risk.

**Summary:**

Preliminary studies demonstrate a significant fraction of airborne particles in cannabis facilities are comprised of fungal spores, bacteria, and plant material, which may also contain hazardous microbial metabolites and allergens. These bioaerosols may pose pathogenic, allergenic, toxigenic, and pro-inflammatory risks to workers. The absence of multi-level, holistic bioaerosol research in cannabis work environments necessitates further characterization of the potential respiratory hazards and effective risk prevention methods to safeguard occupational health as the cannabis industry continues to expand across the US and beyond.

## Introduction

As the cannabis and hemp industries expand across the United States and globally, an increasing number of workers are exposed to respiratory hazards associated with the cultivation and manufacturing of *Cannabis sativa* products. Despite state legalization, the US federal government designates high tetrahydrocannabinol (THC)-containing cannabis as a Schedule I Drug. Therefore, there is no federal oversight of cannabis production by either the United States Department of Agriculture (USDA) or the Food and Drug Administration (FDA). Without these federal crop- and consumer-protection mechanisms, cannabis can be cultivated and processed in environments with potentially increased airborne biological hazards—including but not limited to viable plant and human pathogens, microbial metabolites, toxins, and allergenic proteins—posing health risks to workers, consumers, and plants alike.

Nevertheless, the cannabis market continues to expand in response to state legalization measures. Recently, the Centers for Disease Control (CDC) called for comprehensive measures to protect cannabis workers from inhalation hazards in response to a fatal asthma attack suffered by a worker processing cannabis flower [1]. Undoubtedly, there are large gaps in the scientific basis for providing occupational safety recommendations associated with the potential inhalation hazards unique to indoor cannabis cultivation and manufacturing, as little research has been performed to holistically characterize respiratory risk factors or develop effective prevention measures to control these hazards.

Indeed, it is challenging to parse out meaningful information from the limited body of literature on this topic, as many of the respiratory hazard evaluations in cannabis operations have been performed by state or federal governments in the weeks or months after employee complaints or adverse worker events. Moreover, some reports acknowledge that air sampling was limited as operations were not in full production or that evaluation of air quality was performed after new equipment was installed, such as post-hoc implementation of high-efficiency particulate air (HEPA) filtration [1, 2]. To add more complexity, cannabis work-associated respiratory diseases are likely underreported. This may be due to lack of awareness and training on Occupational Safety and Health Administration (OSHA) protections or due to other cannabis worker concerns about being associated with an industry that is not federally legal. Indeed, a cannabis worker who suffered a fatal asthma attack experienced several respiratory symptoms in the months leading up to their death, including a medical emergency room visit for work-related dyspnea. Moreover, four other co-workers on the associated cannabis flower production team also complained of work-related symptoms, including dyspnea, nasal congestion, and hives. These incidents were not reported until an OSHA investigation surveyed these employees after their co-worker’s death [1]. Holistic investigation of cannabis occupational inhalation hazards is lacking, which in turn prevents this growing industry from better protecting its workers.

In this context, the bioaerosols in cannabis operations are defined as airborne particulate matter comprised in whole or in part of primary biological materials directly associated with all aspects of cannabis operations; these airborne constituents are known to impact worker health in parallel agricultural and manufacturing industries as well as many common indoor environments [3–13]. Bioaerosols remain poorly characterized in the cannabis industry, despite increasing reports of cannabis work-related respiratory diseases linked to bioaerosol exposure [1, 14, 15]. The complexity of bioaerosols, combined with the federal status of state-sanctioned cannabis operations, adds additional barriers for traditional academic research and funding to further investigate cannabis workplace hazards.

In response, this review presents the most current research describing bioaerosols associated with cannabis work environments, outlining the diversity, distribution, and abundance of these airborne constituents, as well as the potential respiratory hazards they pose to workers. We draw from relevant research in indoor and occupational environments, allergy and asthma, and aerobiology of the built environment to investigate respiratory diseases observed in cannabis workers that have potential causal links to related bioaerosol exposures. Where relevant, we will compare bioaerosol exposures observed in parallel industries and environments where indoor air quality (IAQ) and occupational exposure limit (OEL) information is available to provide frame of precedence on the bioaerosol types and exposure levels associated with occupational respiratory diseases.

## Cannabis Cultivation and Bioaerosol Generation

The *C. sativa* plant is divided into two categories based on the abundance of the intoxicating compound THC. This cannabinoid is found within the inflorescence, also known as the flower, of the female plant. Hemp is defined as containing lower levels of THC content by dry weight (< 0.3% THC by weight), while cannabis, often referred to as marijuana, contains higher levels of THC (> 0.3% THC by weight). As such, cannabis is designated as a Schedule I Drug by the Controlled Substances Act and is federally illegal, despite many states legalizing cannabis for medical and recreational use [16, 17].

In addition to differences in cannabinoid content, hemp and cannabis are cultivated and processed to produce distinct end products. Hemp is grown in industrial agricultural settings for its fiber, seed oil, and its non-intoxicating cannabidiol (CBD) content. In contrast, cannabis is grown on a smaller horticultural scale, most often in indoor controlled environment agriculture (CEA) settings to produce flower products and extracts with significantly higher THC content. As such, there is a large diversity of building types and environmental conditions that contain cannabis growing, processing, formulation, and packaging operations [16, 17].

The indoor cannabis CEA environment is optimized for plant growth and flower production, often by regulating temperature, relative humidity (RH), carbon dioxide (CO_2_) levels, and light exposure. Following harvest of mature female plants, cannabis flower is dried and cured to remove excess moisture, followed by processing steps that include milling, trimming, pre-roll manufacturing, and/or extraction of flower materials into cannabinoid-containing oils [16, 17]. Each of these cultivation and manufacturing processes involve different steps that may expose workers to unique respiratory hazards, including exposure to airborne particulate matter (PM).

Airborne PM, comprised either in part or in whole of primary biological materials, makes up a significant portion of PM in the respirable size range (< 10 µm; PM_10_) in both indoor and outdoor settings. Respirable PM is of high concern as it can pass through the nasal passages and deposit into the airways and lungs. Fine PM, smaller than 2.5 µm (PM_2.5_), may directly absorb into the bloodstream from the lung and is linked to several respiratory and systemic diseases, including negative cardiovascular and neurodegenerative effects [18–20]. In addition to differences in respiratory deposition, the indoor settling time also differs depending on the size of these airborne particles. Larger PM settles within seconds to minutes, while finer PM_2.5_ settles in time frames of hours or days, persisting in the work environment. Respiratory exposures are assessed as a combination of the indoor pollutant concentration and the exposure time; thus, respirable bioaerosols may also result in more significant respiratory exposures [21–24].

A common bioaerosol assessment method is to count intact organisms, enumerating fungal spores, pollen granules, culturable colony forming units (CFU), or infectious viral particles within a known volume of air. Modern aerobiology practices can also analyze air samples via DNA- or RNA-based sequencing to identify and estimate the relative abundance of different fungal, bacterial, and viral constituents in the air. Other bioaerosol constituents like microbial metabolites (endotoxin, ergosterol, (1➔3)-β-D-glucan, peptidoglycan, and mycotoxins) and protein allergens are sometimes measured as they are also linked to less well understood mechanisms that contribute to chronic, non-infectious diseases and allergenic respiratory diseases [3–6, 9–11, 21–28].

Bioaerosols are challenging to characterize. While there are strong associations between respiratory exposure to dampness, mold, and organic dusts, direct causation and dose–response data are often lacking. This is partly due to methodological limitations in exposure assessments and to the diverse categories of inhalable biological constituents described above. Bioaerosols also vary temporally and spatially and can have complex interactions with other PM, gasses, humidity, and light in the atmospheric environment [21–24]. Despite these challenges, it is essential to understand bioaerosol size, composition, and abundance, as airborne pathogenic microbes, microbial metabolites, and protein allergens are inhalation hazards associated with a variety of acute and chronic diseases in agricultural and indoor settings that share many aspects with cannabis operations [3–13].

## Bioaerosol Exposure Association with Respiratory Diseases of Cannabis Workers

**Fig. 1:**
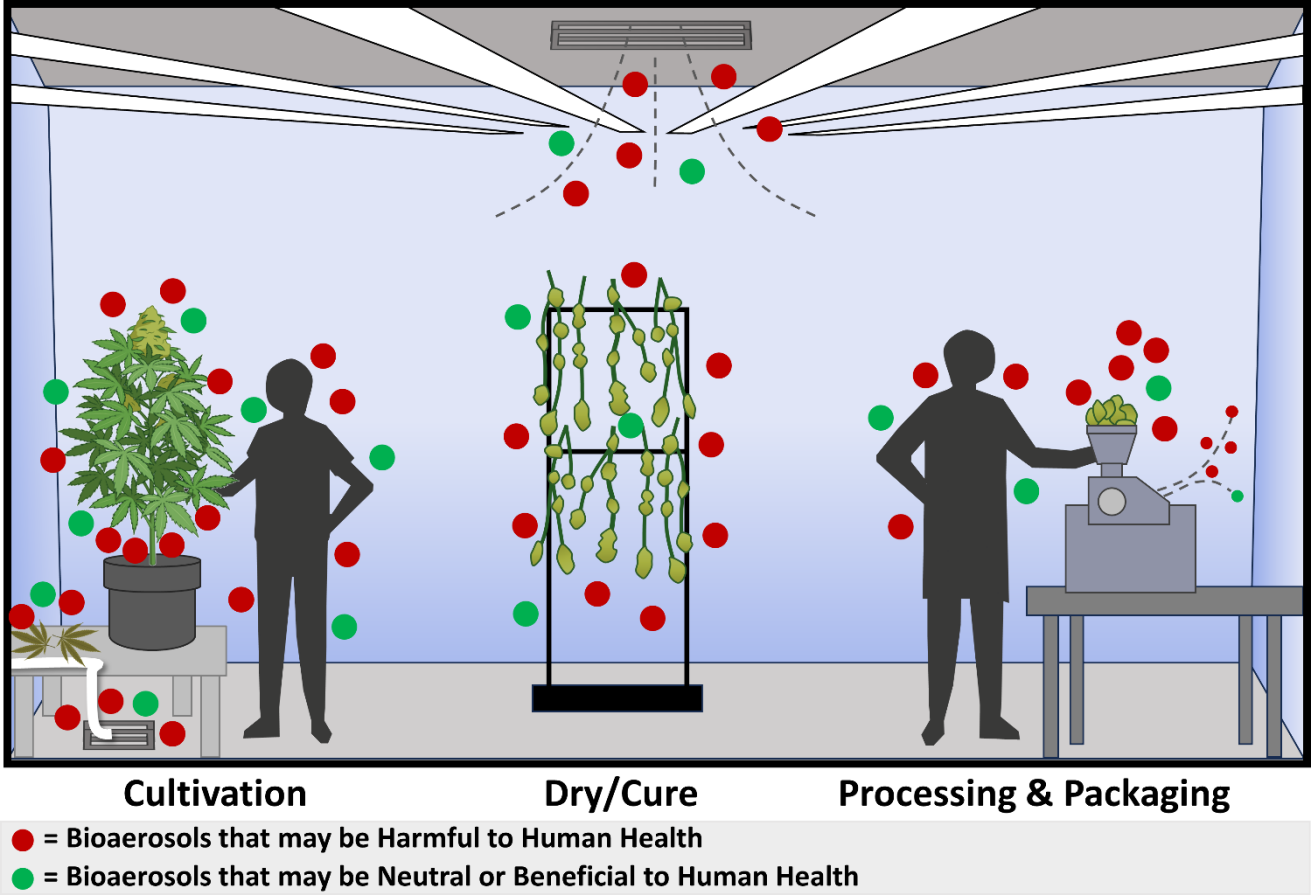
Bioaerosols in cannabis indoor work environments. The cultivation, post-harvest dry/cure, processing, pre-roll manufacturing, and packaging operations all generate unique bioaerosols that vary in composition and size, as well as vary within the operational activity and over time. These airborne biological materials may be harmful, neutral, or potentially beneficial to worker health. Sources for these bioaerosols include cannabis plant matter, microorganisms associated with the plant, building materials, substrates, and post-harvest spoilage, irrigation water and its delivery system, growing substrate and amendments, surfaces, floors, ceilings, walls, HVAC system (ventilation and humidity controllers), and the workers themselves

Unique bioaerosols are present at each stage of the indoor cannabis cultivation and manufacturing process, and exposure may result in neutral, potentially positive, or negative health effects. These bioaerosols likely rapidly cycle in space and time and differ by source generation and operational activity. Indeed, it is likely that the plants and harvested plant matter generate and host much of the bioaerosols associated with indoor cannabis operations; however, there are many other sources as well, including but not limited to the growing substrate, amendments, beneficial organisms, irrigation water and its delivery system, surfaces, floors, ceilings, walls, the HVAC system (both ventilation and humidity controllers), and the workers themselves (Fig. 1).

Causal relationships between bioaerosol exposure and respiratory diseases are difficult to establish. When considering the etiological origins of environmental and occupational lung disease, an important component of disease progression is aberrant immune activation and/or inflammation that is not efficiently resolved, ultimately progressing to tissue injury and/or remodeling that yields the classic hallmarks of diseases such as asthma, chronic obstructive pulmonary disease (COPD), hypersensitivity pneumonitis, and various interstitial lung diseases. As highlighted throughout this review, PM composition is an important determinant to respiratory disease (as well as systemic disease) etiology. For example, inorganic PM (such as black carbon or silica) exposure is often associated with interstitial lung disease pathologies (pneumoconioses) [29, 30], while bioaerosols composed of organic dust PM are commonly associated with allergic, asthmatic, and/or other inflammatory pathologies [10, 11].

At a mechanistic level, the diverse constituents of bioaerosols, including microbial pathogens and their metabolites, as well as allergenic proteins, can drive both inflammation and hypersensitivity responses in exposed individuals. For example, many occupational asthma cases have a clear Type I Hypersensitivity (Immunoglobulin E (IgE)-mediated) response to an allergen in the environment, although some cases are driven by chronic inflammation (without an allergenic component) that leads to bronchoconstriction and tissue remodeling [31–33]. As another example, hypersensitivity pneumonitis is a common interstitial lung disease identified in individuals exposed to certain organic dusts; this immune-mediated process is classically driven by Type III and Type IV Hypersensitivity responses to antigens of bacteria, molds, animals, or low molecular weight chemical compounds [34]. Overall, it is recognized that repeated or chronic exposure to organic dust-derived bioaerosols increases the likelihood for long-term negative respiratory consequences.

**Fig. 2:**
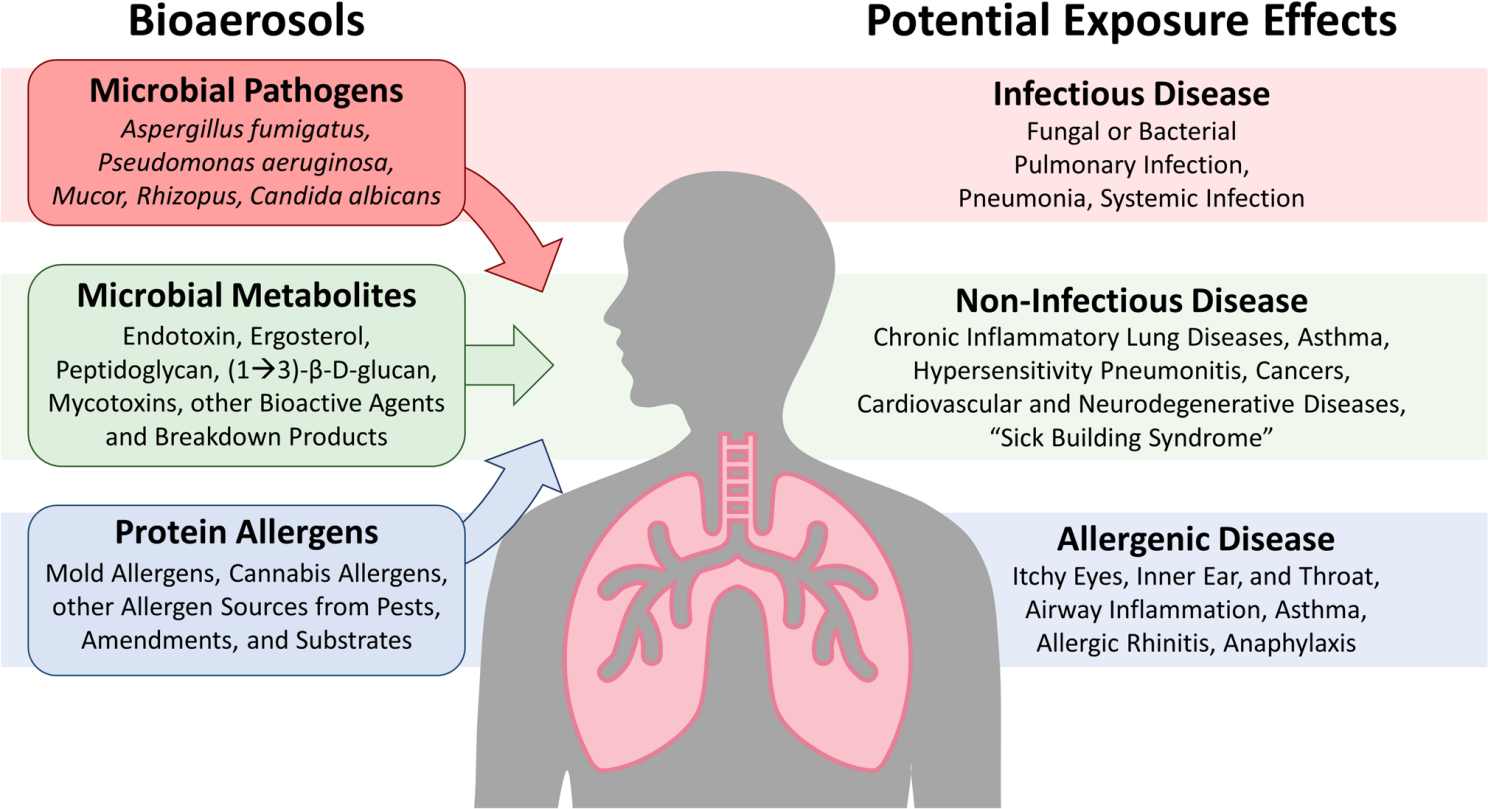
Exposure to complex bioaerosol constituents relevant to cannabis workplaces may contribute to diverse respiratory and systemic effects. Cannabis cultivation and manufacturing facilities harbor airborne bacteria, fungi, plant matter, and other bioaerosol constituents. Airborne pathogens may be inhaled and could result in infection, particularly in susceptible individuals. Other bioaerosol constituents like microbial metabolites and protein allergens are associated with non-infectious and allergenic respiratory diseases. The complex nature of bioaerosols, challenges assessing worker exposure, lack of diagnostic and research tools, and the Schedule I status of cannabis all contribute to the limited data available to directly connect cannabis-relevant bioaerosol exposure to respiratory disease in workers. Therefore, clear causal relationships between bioaerosol exposure and respiratory effects are not fully elucidated

Indeed, bioaerosols linked to disease can contain complex combinations of various inflammatory or otherwise allergenic components that share many similarities with preliminary bioaerosol investigations in cannabis cultivation and manufacturing environments, where bioaerosols have been shown to impact worker safety, plant health, and even regulatory compliance of cannabis flower products [1, 14, 15, 35–42]. Characterization of the airborne PM components in hemp and cannabis workspaces has demonstrated potential for significant levels of bioaerosols, composed of bacteria, fungi, and actinomycetes, as well as high levels of airborne plant matter, protein, and endotoxins—all of which have been associated with a range of health hazards [2, 43–48]. Based on the growing body of research on cannabis- and hemp-related bioaerosols, we categorize bioaerosols that pose inhalation hazards to cannabis workers here as the following: 1) microbial pathogens, 2) toxigenic or pro-inflammatory microbial metabolites, and 3) protein allergens; each of these categories are associated with diverse exposure effects and respiratory diseases (Fig. 2).

## Bioaerosols and their Constituents in Cannabis Work Environments

### Microbial Pathogens

The *C. sativa* microbiome includes many microorganisms, the majority of which are not pathogenic to plants or people; however, there are several toxigenic, allergenic, and/or pathogenic microbial species that have been identified on cannabis plants and flower products [49, 50]. Therefore, these organisms and their metabolites are likely present in cannabis cultivation and manufacturing environments. Studies investigating airborne microorganisms in hemp and cannabis facilities have identified many types of potentially harmful bacterial and fungal microorganisms, including but not limited to Actinobacteria, *Pseudomonas aeruginosa, Staphylococcus, Escherichia coli, Enterobacter, Enterococcus, Botrytis, Aspergillus, Penicillium, Cladosporium, Alternaria alternata*, *Trichoderma viride, Wallemia, Mucor, Rhizopus, Candida albicans,* and *Stachybotrys chartarum* [2, 43–48].

One report estimated levels of airborne mold spores in cannabis facilities at > 500,000 spores/m^3^. When air samples were processed to enumerate airborne CFU, many samples overloaded culture plates, and culturable airborne microorganisms could not be quantified (highest reported as > 11,300 CFU/m^3^). These authors cautioned that elevated mold spore levels warranted respirator use in these environments [43]. Genetic identification of fungal species within cannabis cultivation air samples also showed elevated and enriched mold species typically associated with water-damaged homes [44, 45]. Notably, airborne levels of culturable bacteria and fungi regularly exceeded 1,000,000 CFU/m^3^ within a hemp facility, and workers were provided with respirators for their protection [2]. Although there is evidence that waste collection workers exposed to environmental pathogens are at risk for infectious diseases [51], it is poorly understood how exposure to bioaerosols found in cannabis facilities may cause infection in cannabis workers. Indeed, there is no established OSHA OEL for airborne spores or bacterial/fungal CFU despite the World Health Organization (WHO) categorizing “high” levels of airborne culturable fungal species as ≥ 1,000 CFU/m^3^ [52]. Of note, this WHO categorization is not uniformly accepted in many regulatory circles, and lower levels have been suggested for airborne pathogens and thermophilic actinomycetes as they may be harmful at lower abundance [52–54].

Elevated airborne fungi and bacteria levels in these environments could also contribute to non-pathogenic and allergenic pulmonary diseases within cannabis workers due to respiratory exposures to associated airborne microbial breakdown products, metabolites, and/or allergens, as discussed below [3–13].

### Microbial Metabolites

Endotoxin, also called lipopolysaccharide (LPS), is a macromolecule composed of a lipid and polysaccharide that is found within the Gram-negative bacterial outer membrane. In addition to its functional attributes in bacteria, it can be a potent endotoxin that activates the mammalian immune system and causes inflammation, fever, and septic shock; moreover, endotoxin is an established inhalation hazard linked to inflammatory lung diseases [3, 9–11, 25, 55, 56]. Airborne endotoxin levels are measured as endotoxin units normalized by air volume (EU/m^3^); however, there is no established OSHA OEL for airborne endotoxin. Recently, the Dutch Expert Committee on Occupational Safety (DECOS) proposed an OEL of ≤ 90 EU/m^3^ over an eight-hour weighed average work period, which is used for reference here [57].

There are limited reports measuring airborne endotoxin in hemp and cannabis operations, but they show that endotoxin levels range widely depending on the operational activity and the manufacturing environment. The highest levels of airborne endotoxin in two hemp processing studies were 1,600 EU/m^3^ and 59,801 EU/m^3^, suggesting hemp workers can be exposed to extremely high endotoxin levels [2, 58]. Reports in cannabis operations have generally observed airborne endotoxin below the DECOS exposure limit; however, in two cases endotoxin levels were elevated to 85 or 94 EU/m^3^ during grinding of dried cannabis flower [1, 45, 46, 59].

Importantly, exposure to airborne endotoxin levels below the DECOS limit has been associated with respiratory symptoms and a decline in lung function [56]. This is also acknowledged in the CDC report investigating a fatal cannabis occupational asthma attack, as the authors state, “Airborne respirable dust and endotoxin levels below occupational exposure limits do not exclude a sufficient level of airborne allergen to trigger asthma and other allergic symptoms” [1].

Although airborne endotoxin is a classic industrial hygiene measure, there are a multitude of other airborne microbial metabolites, breakdown products, and associated bioactive agents that may contribute to pulmonary disease following respiratory exposure, several of which are known to contribute to inflammation independent of endotoxin levels [8, 9]. Like bacterial endotoxin, ergosterol is a cell membrane component, and it is important for regulating fungal membrane fluidity. Inhalation exposure to this fungal immunologically active lipid is associated with asthma, and within mammalian cells it can induce pyroptosis, a type of necrotic and inflammatory programmed cell death [8, 25, 60, 61].

Peptidoglycan acts as the backbone of bacterial cell walls. Both Gram-negative and -positive bacteria contain this structural element, but it is present at much higher levels in the latter, and thus is utilized as a surrogate measure of Gram-positive bacteria within dust and air samples. These molecules are recognized by the mammalian innate immune system, inducing a pro-inflammatory response linked to lung inflammation, specifically in response to inhaling organic dusts [9, 62, 63].

The structural element (1➔3)-β-D-glucan is a component of fungal cell walls that is also present in some bacteria and plants. Mammalian phagocytosing cell receptors bind (1➔3)-β-D-glucan molecules, and these fungal molecules elicit pro-inflammatory and cytotoxic responses by disrupting cell cycle signaling, inducing cell death, and causing oxidative stress in vitro. Although broader epidemiological studies are lacking, the presence of (1➔3)-β-D-glucan within occupant air has been associated with airway inflammation and asthma symptoms [26, 27, 64].

Fungal organisms can produce hundreds of mycotoxins, which are low molecular weight molecules that act as virulence factors and defense chemicals. These mycotoxins can cause adverse health effects in humans by inhibiting essential cellular processes, resulting in immunotoxicity, organ-specific toxicity, and cancer [65, 66]. Indeed, parallel agricultural, animal husbandry, and indoor environments have demonstrated that airborne mycotoxins contribute to respiratory diseases as well as “sick building syndrome” [4, 66–68]. Many molds associated with cannabis production have the capacity to produce multiple mycotoxins, including *Alternaria*, *Aspergillus, Penicillium, Stachybotrys, Wallemia,* and *Fusarium* species [36, 43–45, 66, 69]*.*

To the authors’ current knowledge, there are no published studies investigating the abundance of airborne ergosterol, peptidoglycan, (1➔3)-β-D-glucan, or mycotoxins within cannabis work environments or their connection to cannabis worker respiratory disease.

### Protein Allergens

Allergens are most commonly protein macromolecules that activate the mammalian adaptive immune system, initiating a Type I Hypersensitivity (IgE-mediated) response. This IgE response is considered a “classic allergic” response that differs from other pro-inflammatory responses activated by other microbial metabolites described above. Airborne allergen exposure usually causes mild inflammation of the eyes, nose, inner ear, throat, and airways, but these aeroallergens can also have significant health impacts, triggering asthma attacks and even causing anaphylaxis [22, 28, 70].

Because of its complexity, direct aeroallergen monitoring is not commonly performed, and allergen abundance is often indirectly evaluated through surrogates, such as measuring dust levels, culturing airborne bacteria and fungi, microscopic spore counts, or by DNA analysis for target organisms known to produce allergens. Moreover, many bioaerosol collection methods can denature the proteins they are meant to assess; thus, appropriate collection methods must be used to preserve allergen protein structure to allow for accurate immunoassay quantification [22, 28, 70].

To date, data directly identifying and measuring aeroallergens in cannabis facilities are lacking. However, there have been several documented cannabis work-related asthma cases where exposure to airborne mold or plant materials have been contributing factors [1, 14, 15]. Cannabis and hemp work environments can present relatively high levels of airborne protein, organic dusts, molds, and plant matter, suggesting that aeroallergens may be found within these bioaerosols [2, 43–47]. In similar agriculture and CEA environments, allergens and allergenic responses are linked to a diversity of other worksite sources, including spider mite pests, predatory mites, paper pots, and growing substrates like coco coir and rockwool [6, 71–73].

Of the fungi associated with cannabis and cannabis facility air, several are known to produce allergenic proteins, including *Aspergillus, Penicillium, Cladosporium, Alternaria, Rhizopus*, and *Fusarium* species [36, 43–45, 69, 74]. The opportunistic pathogen *Aspergillus fumigatus* was recently designated by WHO as a Critical Priority Fungal Pathogen and is known to produce several mycotoxins; furthermore, *A. fumigatus* also produces at least 30 known allergens listed on the WHO/IUIS (International Union of Immunological Societies) Allergen Nomenclature Database (http://www.allergen.org) [74–76]. This mold species exemplifies the diverse bioaerosol constituents that a single organism can produce, which may cause a wide variety of respiratory exposure responses. In wood milling operations, *A. fumigatus* allergens can be found in wood and wood-derived dust, presenting potential respiratory hazards to workers [77]. Indeed, some allergens are secreted by germinating *A. fumigatus* mycelium and are found associated with spores, so they are likely present on *A. fumigatus-*laden cannabis flower, on growing substrates, and/or building materials in cannabis manufacturing environments, potentially posing an inhalation risk to workers if aerosolized [78].

Studies of some hemp operations found a high prevalence of respiratory symptoms among workers exposed to respirable organic dust, where levels ranged from 10–80 mg/m^3^ [2, 48, 58]. According to OSHA’s Table Z-1-Limits for Air Contaminants, permissible exposure limits to cotton or grain dusts should not exceed levels 1 or 10 mg/m^3^, respectively, over an eight-hour work shift [79], whereas DECOS has recommended a limit of 1.5 mg/m^3^ of inhalable grain dust [80]. These dusts may contain a variety of microorganisms, their metabolites, and protein allergens, including cannabis allergens.

Like many plants, *C. sativa* also produces allergenic proteins, and sensitization to crude cannabis extracts and specific cannabis proteins has been documented in cannabis workers, cannabis consumers, and cannabis laboratory personnel [15, 38–42, 81–84]. In cannabis production environments, the handling and processing of cannabis materials during cultivation and manufacturing likely exposes workers to cannabis allergens, especially during milling and pre-roll manufacturing where aerosolization of dried plant materials often occurs. Indeed, one study found that *C. sativa* DNA sequences made up to 80% of bioaerosol sequencing reads in cannabis worker personal air samples, suggesting plant matter itself composes a large portion of bioaerosols in these environments [45].

Five *C. sativa* allergens are currently listed in the WHO/IUIS Allergen Nomenclature Database: Can s 2, Can s 3, Can s 4, Can s 5, and Can s 7 [76]. Several of these allergens have been covered thoroughly in recent reviews discussing their characteristics, homology to other known plant allergens, and their connection to allergic responses, specifically in cannabis workers [40–42]. Despite the link between cannabis allergen exposure and worker allergic responses, there are limited clinical diagnostic tests to understand cannabis protein mediators of IgE-specific sensitization. Crude cannabis extracts are often used in skin prick tests to determine cannabis-specific allergic sensitization; however, these cannabis extracts are not strictly reproducible, can be difficult to procure in many states, and positive results do not identify the causal allergen(s) involved in IgE-mediated responses. There are Can s 3-based diagnostic tests that detect specific anti-Can s 3 IgE antibodies or patient basophil activation following exposure to purified Can s 3; however, to date, there are no clinically available tests that investigate sensitization to the other four identified cannabis allergens [42, 85].

It is important to emphasize that despite the high levels of plant material suspended in the air of cannabis and hemp facilities, there is no peer-reviewed literature thus far identifying or quantifying airborne cannabis allergens within cannabis workspaces. Moreover, foundational knowledge on cannabis allergens is limited, with little understanding on the tissue-specific expression or chemotype/strain-dependent abundance of cannabis allergens. A recent proteomics investigation of cannabis and hemp flower protein extracts identified 49 potential allergens present across the four different chemotypes tested, including known *C. sativa* allergens Can s 2, Can s 3, and Can s 5; yet Can s 4 was absent in all protein extracts [86]. Indeed, there are large gaps in the basic research of cannabis allergenic proteins, likely due to the limited availability of commercial antibody-based allergen detection methods capable of quantifying these allergens and due to the federal limitations that impede cannabis research under Schedule I classification.

Overall, more diagnostic tool development and associated exposure research is desperately needed to investigate the aeroallergens present within cannabis cultivation and manufacturing environments and connect the presence of these unique aeroallergens to respiratory diseases observed in workers.

## Bioaerosol Synergies

Although we simplify bioaerosols into three categories in Fig. 2, many types of bioaerosols occur simultaneously in the air and several are known to act synergistically to elicit negative health outcomes; therefore, in-depth, multi-level analysis of the diversity of bioaerosols within cannabis environments is needed to understand the multifaced nature of respiratory exposure and associated disease potential. For example, the mycotoxin deoxynivalenol (DON) is produced by the cannabis pathogen *Fusarium*, and it sensitizes mice to protein allergens [36, 64, 87], suggesting it may exacerbate responses to mold or cannabis allergens. Endotoxin can also potentiate the effects of DON, increasing susceptibility of animals and humans to bacterial infections by altering immune responses and enhancing one another’s toxicity [88]. Moreover, general PM exposure in combination with mycotoxins, (1➔3)-β-D-glucan, or allergens can act synergistically to enhance cytotoxicity and inflammation [64, 89, 90]. Thus, it is essential to understand the diverse inhalable risk constituents by developing and implementing meaningful, holistic exposure assessments to better understand the links between diverse, dynamic exposures and disease.

## Management and Prevention

Cannabis industry manufacturers and regulatory bodies should take simple, prophylactic actions to protect workers from potential respiratory hazards, despite the lack of scientific literature on this topic. There are established hierarchy of controls for similar occupational settings to proactively address respiratory hazards [91]. Engineering controls should be implemented to reduce bioaerosol generation and exposure, drawing from effective mitigation strategies that manage other damp, indoor environments [52]. Building materials and substrates should be updated to prevent growth of harmful microorganisms, their metabolites, and allergens. Best practices should be adopted, including appropriate HVAC implementation and enhanced air filtration to control bioaerosols in CEA environments [92].

In a parallel study, worker exposure to airborne fungi, (1➔3)-β-D-glucan, bacteria, endotoxin, and dust in vegetable greenhouses was higher when handling dried plant materials versus freshly harvested material [93]. This demonstrates how simple aerosol management measures, including HEPA air filtration, should be a top priority in cannabis cultivation, processing, pre-roll manufacturing, and packaging to reduce overall bioaerosol levels, with a specific focus on post-harvest handling and processing of dried plant materials. Cost-effective air quality monitoring tools are available that measure PM_10_ and PM_2.5_, allowing real-time evaluation of respirable PM and verification of the effectiveness of these control measures. Personal protective equipment (PPE) and respiratory protection programs may be required if other steps within the hierarchy of controls do not effectively control these hazards. Worker education and training programs should also be standardized to inform workers about the bioaerosol exposure risks in cannabis work environments, how to report any adverse health effects, and how to best protect themselves. Expansion and more consistent implementation of these programs is needed across the US.

## Conclusions

Bioaerosol research in cannabis cultivation and manufacturing is in its early stages, but the connection between bioaerosol exposure and worker respiratory diseases is concerning. There is a large gap in the scientific literature characterizing bioaerosols that may pose inhalation hazards to cannabis workers–no comprehensive or unified approach yet exists. There is a great need to understand airborne microorganisms in these environments, as well as their aerosolized pro-inflammatory and toxic metabolites, including but not limited to the prevalence of airborne endotoxin, ergosterol, (1➔3)-β-D-glucan, peptidoglycan, and mycotoxins. Aeroallergens produced within the cannabis cultivation and manufacturing environments are also poorly understood, specifically those allergens produced by the *C. sativa* plant itself.

Unfortunately, the proposed re-scheduling of cannabis to a Schedule III Drug [94] will not likely alleviate the research restrictions on studying non-federally recognized, but state-sanctioned, cannabis operations and products, especially if research samples may contain THC. Federal regulatory barriers must be removed from this “gray area” between federal and state legalities, and specific funding could be made available for researchers to identify and quantify the types of bioaerosols present in cannabis occupational settings, assess worker exposure levels, and investigate the link between exposure and respiratory diseases. Only after the scientific community establishes foundational knowledge of the multi-level and diverse bioaerosol constituents in these environments, can evidence-based exposure levels be leveraged to develop meaningful occupational exposure guidelines.

Overall, a proactive, multi-level research approach investigating the abundance and diversity of bioaerosols in cannabis work environments is critical to understand and control the health risks to workers. By taking these steps, we can use evidence-based methods to better protect cannabis workers from the harmful effects of bioaerosol exposures and help ensure a safe and healthy work environment for this expanding industry.

## Data Availability

No datasets were generated or analysed during the current study.
